# Prevalence of Lifestyle Factors Among Primary Care Physicians: A Cross-Sectional Study in Al-Ahsa, Saudi Arabia

**DOI:** 10.7759/cureus.67900

**Published:** 2024-08-27

**Authors:** Wejdan H Alqatifi, Abdulkareem J Alquwaidhi, Rahma B AlGadeeb

**Affiliations:** 1 Preventive Medicine Department, Al-Ahsa Health Cluster, Ministry of Health, Al-Ahsa, SAU

**Keywords:** physical activity, caffeine, smoking, obesity, al-ahsa, saudi arabia, prevalence, primary healthcare center, primary care physicians, lifestyle factors

## Abstract

Introduction

The importance of a healthy lifestyle has grown in significance on a global scale, as it offers a vital means of preventing and managing a range of related illnesses. Consequently, maintaining a healthy lifestyle is of paramount importance for the prevention and management of non-communicable diseases. The lifestyle behaviors of healthcare workers exert a significant influence on their attitudes and counseling methods, as they play a pivotal role in the promotion of health and the dissemination of lifestyle education to patients and the general population.

Objective

The objective of this study was to examine the prevalence of lifestyle factors among primary care physicians in Al-Ahsa Governorate, including smoking, body mass index, nutrition, physical activity, and caffeine consumption.

Methods

The study was conducted between December 2023 and February 2024. A total of 233 primary care physicians in Al-Ahsa were selected through a probability multistage clustering sampling method. Data were collected via the distribution of a self-administered questionnaire to the primary care physicians and were analyzed using the chi-square test.

Results

A greater proportion of primary care physicians exhibited multiple unhealthy lifestyle factors (166/233, 71.2%). The most prevalent lifestyle factor was low physical activity (169/233, 73%), followed by poor nutrition (121/233, 52%), obesity (120/233, 51.51%), smoking (37/233, 15.88%), and caffeine consumption (22/233, 9%). The majority of primary care physicians with optimal health status are employed in primary healthcare (PHC) facilities situated in the eastern region followed by the southern region in Al-Ahsa Governorate.

Conclusion

The study findings revealed a prevalence of unhealthy lifestyle factors among the majority of primary care physicians in the Al-Ahsa Governorate. The most prevalent unhealthy lifestyle factor among the participants was low physical activity.

## Introduction

Unhealthy behaviors and lifestyles represent the primary modifiable risk factors for noncommunicable diseases (NCDs), collectively accounting for the majority of the global disease burden [1,2]. Factors such as overweight, poor nutrition, smoking, alcohol consumption, and low physical activity have been identified as significant contributors to the development of NCDs, including cancer, diabetes, and cardiovascular disease (CVD) [1,3,4]. The World Health Organization (WHO) has estimated that the implementation of appropriate interventions could potentially avert 39 million deaths by 2030 [5].

Lifestyle-related disorders are a significant contributor to the rising costs of healthcare, as well as an increasing number of fatalities and instances of morbidity on a global scale. In England, for instance, CVDs have been identified as the underlying cause of over four million disabilities and 150,000 deaths [6].

Healthcare experts have highlighted the significance of adopting a healthy lifestyle, particularly with regard to diet, as a means of reducing the risk of developing CVD in the future [7]. The United Kingdom experienced a 68% reduction in CVD-related mortality from 1980 to 2013 [8]. Americans have a lower life expectancy compared to other high-income countries; however, adopting a healthy lifestyle could increase life expectancy and reduce premature mortality [9]. The WHO recommends a healthy diet, a tobacco-free lifestyle, regular physical activity, and an environment that promotes social and emotional well-being [10].

The WHO underscores the significance of adopting a healthy lifestyle as a means of preventing chronic diseases, collectively known as NCDs [11]. The principal risk factors for NCDs include tobacco use, physical inactivity, alcohol consumption, an unhealthy diet, and air pollution [12]. The Gulf Cooperation Council (GCC) countries are most affected by risk factors for lifestyle diseases such as diabetes, CVD, and obesity [13,14].

The increase in obesity and diabetes in the GCC is attributed to a lack of health knowledge, negative attitudes, and poor lifestyle choices. It is recommended that primary care physicians receive training in smoking cessation programs [15,16]. In the GCC, for instance, NCDs account for 69% to 83% of all deaths [13]. Moreover, the GCC countries are ranked first globally in terms of risk factors for lifestyle diseases, including diabetes, CVD, and obesity [14]. For instance, Saudi Arabia exhibits the highest prevalence of diabetes (20.5%), followed by Qatar (16.3%), Kuwait (17.9%), Bahrain (17.5%), the UAE (10.7%), and Oman (8.2%) [14].

Internationally, studies have shown varying levels of unhealthy lifestyle behaviors among health professionals. For example, a study in Poland found that only 11% of general practitioners met the criteria for a healthy lifestyle [17]. Some studies have reported that health workers in Malaysia have a higher risk of obesity compared to the general adult population [18]. A recent study in Spain reported higher alcohol consumption among physicians compared to the general population [19]. At the regional level, a study in Iraq reported a prevalence of tobacco use of 33.33% among healthcare workers in primary healthcare centers (PHCs) in the city of Mosul [20]. Although physicians are supposed to recommend healthy behaviors to their patients, they themselves do not always meet these criteria, as previously reported in a study conducted in Bahrain [21].

Although a healthy lifestyle is of paramount importance for all individuals, a substantial proportion of the literature exclusively concentrates on the role of patients and neglects to acknowledge the pivotal part played by healthcare professionals in promoting and sustaining a healthy lifestyle. It is imperative that healthcare personnel exemplify leadership through their own actions, engaging in physical exercise, quitting smoking, and consuming healthy foods. Fundamentally, healthcare providers have a professional obligation to offer the best possible care to patients. In this context, part of their responsibilities includes advising people on how to eat healthily, live longer, and be mentally competent to deal with daily obstacles. However, there are instances when healthcare professionals are unable to fulfill this obligation due to various challenges [22].

In the Kingdom of Saudi Arabia, there is a discrepancy between the training demands of primary healthcare professionals and the healthcare practices they employ, as well as a discrepancy between the efficacy of self-perceived treatments and the efficacy of recommended standards of care. Additionally, there is a discrepancy between recommended standards and practices, and a lack of resources [23].

It has been demonstrated that the lifestyle habits of healthcare providers can influence their attitudes and counseling approaches toward patients. Healthcare professionals play a pivotal role in promoting health and providing lifestyle education to patients and the public [24].

The objectives of this study were to assess the prevalence of unhealthy lifestyle factors among primary care physicians in Al-Ahsa, Saudi Arabia. Specifically, the study aimed to assess the presence of smoking, poor diet, low physical activity, high body mass index, and unhealthy caffeine consumption. These factors were explored using validated questionnaires to measure their prevalence among a representative sample of 233 primary care physicians. Targets were set to ensure recruitment and data analysis within the timeframe of December 2023 to February 2024. This study provides insights into the lifestyle behaviors of primary care physicians that may influence their health promotion practices and patient counseling.

## Materials and methods

This cross-sectional analytical study was conducted in PHCs in Al-Ahsa, Eastern Province, Saudi Arabia. The study population consisted of primary care physicians who have been working in a clinic for at least one year since they were hired. It was conducted between December 2023 and February 2024.

The sample size was determined based on the following formula for sample size calculation: N = [Z^2^ X *p* X (1 - *p*)] / *e*^2^.

where N is the required sample size, Z is the Z-score (1.96 for a 95% confidence level), *p* is the estimated prevalence, and *e* is the margin of error (5%). For this study, the following values were used: Z = 1.96 (corresponding to a 95% confidence level), *p* = 0.5 (assuming a prevalence of 50% for maximum sample size), and *e* = 0.05 (margin of error). Therefore, N = 384.16. After applying the finite population correction for the total population of primary care physicians in Al-Ahsa, the sample size was adjusted. This resulted in a sample size of 194 participants. To account for potential nonparticipation and incomplete responses, an additional 20% (39 participants) was added to the calculated sample size, resulting in a final total of 233 participants. The assumed prevalence rate of 50% is commonly used in the absence of specific prevalence data to maximize sample size.

A total of 233 primary care physicians in Al-Ahsa were recruited using probability multistage cluster sampling. Al-Ahsa is divided into four health sectors: South, Central, North, and East, with 18, 11, 16, and 19 PHCs, respectively. In the first stage, PHCs from each sector were selected using a simple random sampling technique. In the second stage, primary care physicians working in the selected PHCs were randomly recruited to participate in the study.

The study employed a representative sample of primary care physicians in Al-Ahsa City to evaluate the distribution and prevalence of various health risk behaviors through the use of an anonymous, self-administered, paper-based questionnaire. The target population consisted of primary care physicians, with inclusion and exclusion criteria applied to ensure eligibility. In particular, the study included only physicians who had been in practice for at least one year, while those who had not met this criterion were excluded from the study.

The data were collected using the following previously validated questionnaires:

The subject's physical activity level was determined through the administration of the International Physical Activity Questionnaire - Short Form (IPAQ-SF), which was established in Geneva in 1998 and validated across 12 countries. Physical activity was deemed healthy if participants engaged in at least 150 minutes of moderate-intensity aerobic activity or 75 minutes of vigorous-intensity activity per week, while those engaging in less were classified as unhealthy [25].

The subject's smoking habits were assessed using the Tobacco Use Among Health Care Workers Questionnaire, which was previously utilized in research conducted in Abha City. This questionnaire is based on a WHO questionnaire and relevant literature, and it was approved by the King Khalid University Ethical Committee. Nonsmokers and former smokers who quit more than six months ago were considered healthy, while current smokers were classified as unhealthy [26].

In terms of dietary habits, the evaluation was conducted using the Food Frequency Questionnaire, which was employed in a study conducted in Gothenburg from 2014 to 2016 and is recommended by the Nordic Nutrition Recommendations. A healthy diet includes at least five servings of fruits and vegetables per day and a low intake of processed foods and sugars, while the opposite is considered unhealthy [27].

The quantity of caffeine consumed was determined using the Caffeine Consumption Questionnaire-Revised. Individuals who consumed 400 mg or less of caffeine per day were categorized as having a healthy caffeine consumption, while those who consumed more than this amount were categorized as having an unhealthy caffeine consumption [28].

Participants were required to report their BMI category; those who were unsure of their BMI range were excluded from the study. A healthy BMI ranged from 18.5 to 24.9 kg/m², with underweight (<18.5 kg/m²), overweight (≥25.0 kg/m²), or obese (≥30.0 kg/m²) BMIs being classified as unhealthy.

The questionnaire was distributed manually by the researcher to the participants in each selected PHC facility. The responses to the questionnaires were randomly selected to be included in the data set. Participants were duly informed of the purpose of the study, the manner in which the data would be collected, and their consent was obtained. They were also assured of the confidentiality of the data. The majority of health-related lifestyle factors (study variables) identified by the WHO as being of concern, including being overweight or obese, poor nutrition (regular consumption of large amounts of processed foods, sugary beverages, and foods high in unhealthy fats, while having a low intake of fruits, vegetables, whole grains, and lean proteins), smoking, low levels of physical activity, and alcohol consumption (replaced by caffeine intake in this study), were the primary focus of the research. The study was conducted in accordance with the ethical standards set forth by the IRB of King Fahad Hospital - Al Hofuf (14-E-23).

The data analysis was conducted using IBM SPSS Statistics for Windows, Version 25 (Released 2017; IBM Corp., Armonk, New York). The results are presented as means and standard deviations for continuous variables and as frequencies and percentages for categorical variables. The statistical analyses used chi-square tests for categorical data. A p-value of less than 0.05 was used to determine statistical significance.

## Results

A total of 233 physicians were included in the calculated sample. Demographic information and categorical frequencies indicated that 141 (60.52%) participants were male, while 92 (39.49%) were female. The age group with the highest representation was that of individuals between the ages of 31 and 36. With regard to marital status, 186 (79.83%) participants were married, 42 (18.03%) were single, and 5 (2.15%) were divorced. Most participants (147/233, 63.1%) had at least five years of experience working in a primary healthcare center. The physicians were randomly selected from each sector based on the previously computed total number of physicians in each sector (Table 1).

**Table 1:** Characteristics of Primary Care Physicians.

Factor		N (%)
Gender	Female	92 (39.49%)
	Male	141 (60.52%)
Age	25-30	58 (24.9%)
	31-36	94 (40.35%)
	37-43	49 (21.04%)
	44-49	20 (8.59%)
	50-55	12 (5.16%)
Marital status	Single	42 (18.03%)
	Married	186 (79.83%)
	Divorced	5 (2.15%)
Working in a primary healthcare center	1-2 years	43 (18.46%)
	2-3 years	21 (9.02%)
	3-4 years	22 (9.45%)
	≥5 years	147 (63.1%)
Sector	Middle	58 (24.9%)
	Northern	61 (26.19%)
	Southern	51 (21.89%)
	Eastern	63 (27.04%)

Table 2 presents the health status of individuals based on five factors: smoking status, BMI, physical activity, caffeine consumption, and adherence to a healthy diet. Each factor is classified as either "healthy" or "unhealthy," with corresponding frequencies and percentages. The categorization was based on whether the mean score was greater than or equal to 0.05, indicating a healthy status, and less than 0.05, indicating an unhealthy status.

**Table 2:** Overall Health Status by Each Factor. *The Overall Health Score is calculated based on the classification of five primary factors. If four or five of these factors are classified as "healthy," the overall health score is deemed "healthy." Conversely, if three or fewer of these factors are categorized as "healthy," the overall health score is considered "unhealthy."

Factor	Unhealthy	Healthy	Mean	Direction
	N (%)	N (%)		
Smoking	37 (15.88%)	196 (84.13%)	0.841	Healthy
Body mass index	120 (51.51%)	102 (43.78%)	0.459	Unhealthy
Physical activity	169 (73%)	64 (27%)	0.292	Unhealthy
Caffeine consumption	22 (9%)	211 (91%)	0.9	Healthy
Healthy diet	121 (52%)	112 (48%)	0.48	Unhealthy
Overall health score*	166 (71.2%)	67 (28.8%)	0.287	Unhealthy

A significant gender difference was observed in smoking status (p = 0.015), with a higher percentage of males (112, 48.1%) being classified as healthy compared to females (84, 36.1%). No significant gender differences were observed for BMI (p = 0.480), physical activity (p = 0.200), caffeine consumption (p = 0.547), healthy diet (p = 0.835), or overall health score (p = 0.467) (Table 3).

**Table 3:** Healthy Lifestyle Factors Among Primary Care Physicians by Gender. *Significant at p-value less than 0.05.

Factor	Gender	Unhealthy	Healthy	Total	P-Value
Smoking	Female	8 (3.4%)	84 (36.1%)	92 (39.5%)	0.015^*^
	Male	29 (12.4%)	112 (48.1%)	141 (60.5%)	
	Total	37 (15.9%)	196 (84.1%)	233 (100%)	
Body mass index	Female	45 (20.3%)	43 (19.4%)	88 (39.6%)	0.480
	Male	75 (33.8%)	59 (26.6%)	134 (60.4%)	
	Total	120 (54.1%)	102 (45.9%)	222 (100%)	
Physical activity	Female	71 (30.5%)	21 (9%)	92 (39.5%)	0.200
	Male	98 (42.1%)	43 (18.5%)	141 (60.5%)	
	Total	169 (72.5%)	64 (27.5%)	233 (100%)	
Caffeine consumption	Female	10 (4.3%)	82 (35.19%)	92 (39.5%)	0.547
	Male	12 (5.2%)	129 (55.4%)	141 (60.5%)	
	Total	22 (9.4%)	211 (90.6%)	233 (100%)	
Healthy diet	Female	47 (20.2%)	45 (19.3%)	92 (39.5%)	0.835
	Male	74 (31.8%)	67 (28.8%)	141 (60.5%)	
	Total	121 (51.9%)	112 (48.1%)	233 (100%)	
Overall health score	Female	68 (29.2%)	24 (10.3%)	92 (39.5%)	0.467
	Male	98 (42.1%)	43 (18.5%)	141 (60.5%)	
	Total	166 (71.2%)	67 (28.8%)	233 (100%)	

The data revealed a significant influence of age on several factors related to health status, including BMI (p = 0.029), physical activity (p = 0.01), and healthy diet (p = 0.011). Conversely, no significant association was observed between age groups and smoking status (p = 0.291), caffeine consumption (p = 0.181), or overall health score (p = 0.087) (Table 4).

**Table 4:** Healthy Lifestyle Factors Among Primary Care Physicians by Age. *Significant at p-value less than 0.05. "44+" was formed by merging "44-49" with "50-55" to meet chi-square assumptions due to fewer than five samples in some categories.

Factor	Age	Unhealthy	Healthy	Total	P-Value
Smoking	25-30	8 (3.4%)	50 (21.5%)	58 (24.9%)	0.291
	31-36	19 (8.21%)	75 (32.2%)	94 (40.3%)	
	37-43	8 (3.4%)	41 (17.59%)	49 (21%)	
	44+	2 (0.91%)	30 (12.9%)	32 (13.7%)	
	Total	37 (15.9%)	196 (84.1%)	233 (100%)	
Body mass index	25-30	23 (10.4%)	34 (15.29%)	57 (25.7%)	0.029*
	31-36	46 (20.7%)	42 (18.9%)	88 (39.6%)	
	37-43	31 (14.02%)	16 (7.2%)	47 (21.2%)	
	44+	20 (9%)	10 (4.5%)	30 (13.5%)	
	Total	120 (54.1%)	102 (45.9%)	222 (100%)	
Physical activity	25-30	33 (14.2%)	25 (10.7%)	58 (24.9%)	0.01*
	31-36	69 (29.59%)	25 (10.7%)	94 (40.3%)	
	37-43	40 (17.2%)	9 (3.9%)	49 (21%)	
	44+	27 (11.6%)	5 (2.1%)	32 (13.7%)	
	Total	169 (72.5%)	64 (27.5%)	233 (100%)	
Caffeine consumption	25-30	8 (3.4%)	50 (21.5%)	58 (24.9%)	0.181
	31-36	10 (4.3%)	84 (36.1%)	94 (40.3%)	
	37-43	4 (1.7%)	45 (19.3%)	49 (21%)	
	44+	0 (0%)	32 (13.7%)	32 (13.7%)	
	Total	22 (9.4%)	211 (90.6%)	233 (100%)	
Healthy diet	25-30	40 (17.2%)	18 (7.7%)	58 (24.9%)	0.011*
	31-36	41 (17.59%)	53 (22.7%)	94 (40.3%)	
	37-43	27 (11.6%)	22 (9.4%)	49 (21%)	
	44+	13 (5.6%)	19 (8.2%)	32 (13.7%)	
	Total	121 (51.9%)	112 (48.1%)	233 (100%)	
Overall health score	25-30	38 (16.3%)	20 (8.6%)	58 (24.9%)	0.087
	31-36	62 (26.6%)	32 (13.7%)	94 (40.3%)	
	37-43	39 (16.7%)	10 (4.3%)	49 (21%)	
	44+	27 (11.6%)	5 (2.1%)	32 (13.7%)	
	Total	166 (71.2%)	67 (28.8%)	233 (100%)	

## Discussion

To the best of our knowledge, no previous study has examined lifestyle factors among primary care physicians in Al-Ahsa. Furthermore, there is no research in Saudi Arabia on lifestyle variables affecting primary care physicians [29]. Primary care physicians can improve the health of the population by providing preventive care, counseling, and interventions. Their influence as "social models" may be modified by their health-related behaviors [30]. Physicians who practice what they preach not only improve their own health but also act as better advisors to their patients and are more effective at counseling [24,30,31].

Smoking among primary care physicians is detrimental to their health and may influence their attitudes toward smoking cessation counseling [16]. In Iraq, a descriptive study was conducted to determine the prevalence of tobacco use among health professionals in PHCs of Mosul City, which was found to be 33.33% [20]. An earlier study in Saudi Arabia, using self-reported questionnaires distributed to health workers in government hospitals and PHCs in Abha, found a similar proportion (26.3%) to be smokers [32]. In Catalonia, a study was conducted on the physical activity levels of primary healthcare workers who spontaneously attended a medical examination. Primary healthcare workers have different levels of physical activity than the general population. Unfortunately, they did not have enough valid data for comparison [33].

In the Jouf region of Saudi Arabia, a study conducted in 2015 found that the majority of PHCs in the cities of Sakaka and Dumat Al-Jandal were physically active and able to provide healthy behavior counseling to their patients. Among physically inactive primary care physicians, there was significant motivation to increase physical activity [34].

Unhealthy eating habits and lifestyles have a negative impact on health, with 32% of health professionals surveyed in China 2020 across 31 provinces, cities, and municipalities being overweight or obese [10].

The literature on healthy lifestyles often overlooks the role of healthcare professionals in maintaining healthy lifestyles. Healthcare providers have a professional obligation to provide patients with the best possible care, including advice on healthy eating, longevity, and mental competence [22]. However, there are gaps in training, healthcare practices, self-perceived treatment effectiveness, discrepancies between recommended standards, and a lack of resources in Saudi Arabia. Primary care physicians play a critical role in promoting healthy lifestyles and educating patients and the public [23]. The lifestyle habits of healthcare providers have been shown to influence the attitudes and counseling approaches of patients. Healthcare professionals have an essential role in health promotion and lifestyle education for patients and the general public [24].

Healthcare professionals who lead healthy lifestyles are better equipped to promote health literacy among patients [31]. However, studies show that health professionals are not more likely to participate in worksite health promotion (WHP) programs, which are essential for their health and ability to counsel patients [24]. In Poland, only 11% of general practitioners meet the criteria for a healthy lifestyle, while 30% of primary care physicians are high-risk drinkers and smokers [30]. This increases the risk of poor quality of life for health professionals, including family physicians in Saudi Arabia [35].

In this context, our study aimed to assess lifestyle factors, such as smoking, BMI, nutrition, physical activity, and caffeine consumption among primary care physicians in Al-Ahsa. By analyzing these factors, this study seeks to contribute to the understanding of lifestyle influences on NCDs and offer valuable recommendations to relevant authorities.

Implementing treatments and approaches to improve physicians' well-being and work situations is critical and should be included early in the medical curriculum [30]. In this context, health professionals who live healthy lifestyles are better able to promote health literacy among patients [30,31]. Health literacy is important for both individuals and health professionals. Healthcare institutions where health professionals study, practice and eventually work must provide opportunities for health promotion and healthy lifestyle aspects [36]. In Saudi Arabia, interventions and services typically focus on behavior modification to address specific risk factors. Unfortunately, no previous studies have been conducted in Al-Ahsa regarding the prevalence of healthy lifestyle risk factors among primary care physicians. This study demonstrates the need to address multiple risk factors in policies and interventions among primary care physicians in Al-Ahsa Governorate.

There are several limitations to this study. First, the cross-sectional design limits the ability to infer causality between lifestyle factors and health outcomes among primary care physicians. Second, the self-reported nature of the questionnaires may introduce reporting bias, as participants may overestimate or underestimate their behaviors. Third, the study focused only on primary care physicians in Al-Ahsa, limiting the generalizability of the findings to other regions or health care professionals in Saudi Arabia. Finally, the exclusion of physicians with less than one year of practice may have overlooked lifestyle factors affecting less experienced physicians.

## Conclusions

This study provides valuable insights into the lifestyle factors of primary care physicians in Al-Ahsa. The findings underscore the importance of promoting healthy lifestyles among healthcare providers, whose behaviors may influence their effectiveness in counseling patients and promoting public health. Addressing these lifestyle factors through early inclusion in medical curricula and workplace health promotion programs could improve physicians' well-being and their ability to serve as role models for healthy behaviors. This study contributes to the literature on lifestyle and NCDs and provides recommendations for health authorities to implement comprehensive strategies to improve the health and working conditions of primary care physicians.
